# Somatostatin receptors in congenital hyperinsulinism: Biology to bedside

**DOI:** 10.3389/fendo.2022.921357

**Published:** 2022-09-27

**Authors:** Mirjam E. van Albada, Klaus Mohnike, Mark J. Dunne, Indi Banerjee, Stephen F. Betz

**Affiliations:** ^1^ Department of Paediatric Endocrinology, University Medical Center Groningen, University of Groningen, Groningen, Netherlands; ^2^ Universitätskinderklinik, Otto-von-Guericke-Universität, Magdeburg, Germany; ^3^ Department of Physiology, Faculty of Biology, Medicine and Health, University of Manchester, Manchester, United Kingdom; ^4^ Department of Paediatric Endocrinology, Royal Manchester Children’s Hospital and Faculty of Biology, Medicine and Health, University of Manchester, Manchester, United Kingdom; ^5^ Crinetics Pharmaceuticals, San Diego, CA, United States

**Keywords:** congenital hyperinsulinemia, somatostatin, receptor expression, hypoglycemia, insulin, glucagon

## Abstract

Congenital hyperinsulinism (CHI), although a rare disease, is an important cause of severe hypoglycemia in early infancy and childhood, causing preventable morbidity and mortality. Prompt diagnosis and appropriate treatment is necessary to prevent hypoglycaemia mediated brain damage. At present, the medical treatment of CHI is limited to diazoxide as first line and synthetic somatostatin receptor ligands (SRLs) as second line options; therefore understanding somatostatin biology and treatment perspectives is important. Under healthy conditions, somatostatin secreted from pancreatic islet δ-cells reduces insulin release through somatostatin receptor induced cAMP-mediated downregulation and paracrine inhibition of β- cells. Several SRLs with extended duration of action are now commercially available and are being used off-label in CHI patients. Efficacy remains variable with the present generation of SRLs, with treatment effect often being compromised by loss of initial response and adverse effects such as bowel ischaemia and hepatobiliary dysfunction. In this review we have addressed the biology of the somatostatin system contexualised to CHI. We have discussed the clinical use, limitations, and complications of somatostatin agonists and new and emerging therapies for CHI.

## Somatostatin perspectives in congenital hyperinsulinism

Congenital hyperinsulinism is a rare disease with a significant genetic component causing unregulated overproduction of insulin through defects in the insulin-releasing pathway. The coupling of insulin secretion to glucose concentration is not tightly regulated, leading to episodes of severe and recurrent hypoglycemia. Several causative mutations in multiple genes have been described to date. The most severe forms are caused by recessive mutations in *ABCC8* and *KCNJ11* coding for the subunits SUR1 and Kir6.2 of the ATP-sensitive K+ (K-ATP) channel on the pancreatic β-cell membrane. Homozygous and compound heterozygous mutations, as well as dominantly inherited mutations in *ABCC8*/*KCNJ11* cause diffuse CHI, which is often unresponsive to first line diazoxide treatment and therefore suitable for therapy with second line somatostatin receptor ligands (SRLs). In contrast, a subgroup of patients with paternally inherited recessive mutations in *ABCC8/KCNJ11* may have focal disease, potentially curable by surgical excision of a solitary lesion.

Excess insulin in CHI precludes the development of ketones. Therefore, in CHI there is the absence of both glucose and ketones as primary and alternative fuel sources for brain cells, leading to brain damage. In the short term, intravenous high concentration dextrose and in some patients, continuous administration of feeds with a high carbohydrate content are used to prevent hypoglycemia. Excess carbohydrate administration has the propensity to cause obesity and interfere with the normal development of oral feeding. In many such patients, diazoxide is ineffective, resulting in the need for a trial of SRL to reduce excess insulin secretion, before resorting to irreversible sub-total pancreatectomy with consequent development of insulin dependent diabetes and exocrine pancreatic insufficiency.

## Biology of the somatostatin system

**Figure 1:**
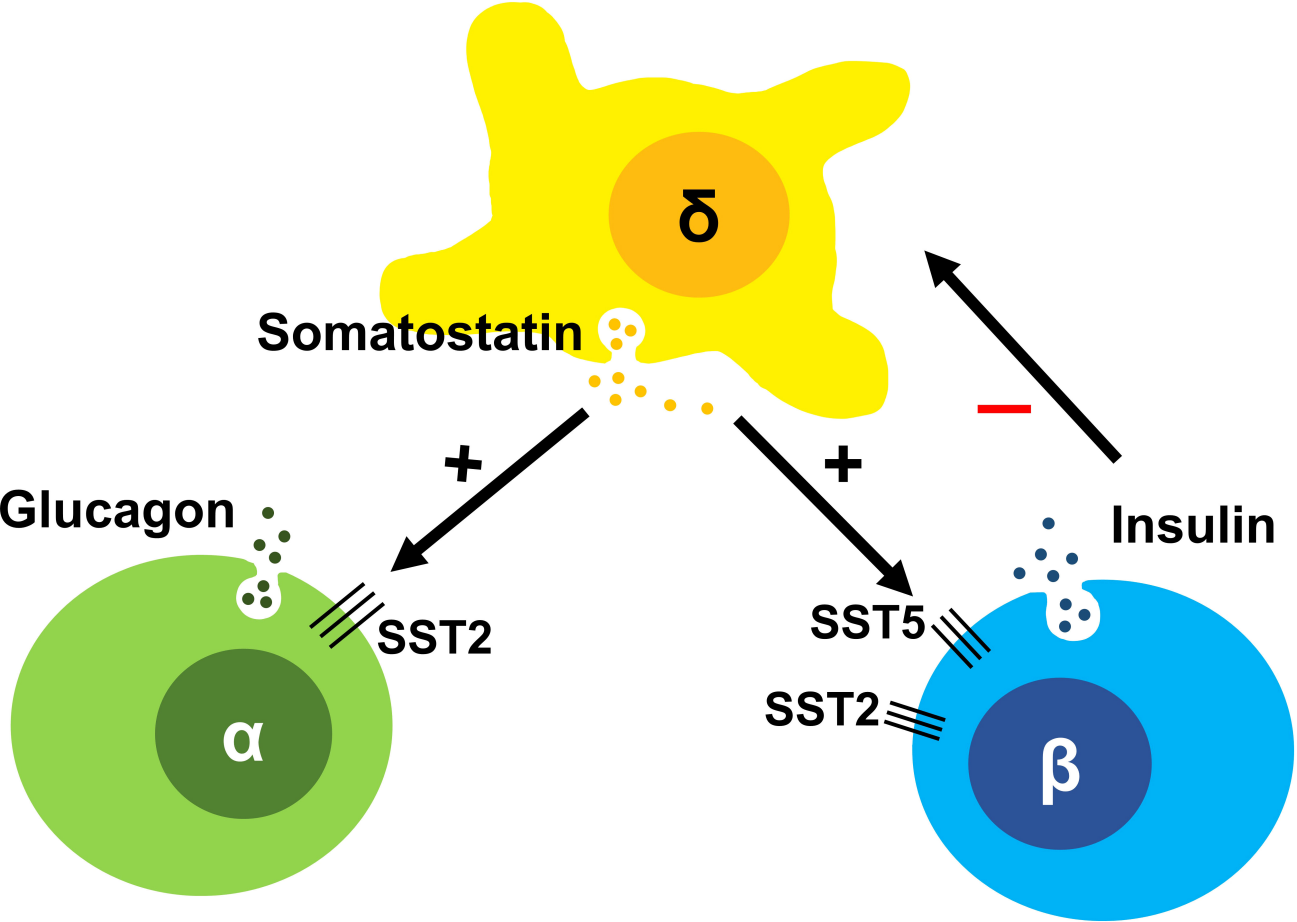
The somatostatin induced inhibitory paracrine regulation of the islet system comprising of α, β and δ-cells, with δ-cells inhibiting sustained insulin secretion and so preventing hyperinsulinism in the normal state.

Somatostatin is a peptide hormone first isolated from the ovine hypothalamus and noted to be a somatotroph release-inhibiting factor (SRIF) (1, 2). Outside the central nervous system, somatostatin is also produced in δ-cells of the pancreas, in close proximity to α- and β-cells (3, 4), setting up opportunities for fine paracrine regulation of both insulin and glucagon secretion (5, 6), **Figure 1**. In the gut, somatostatin inhibits in particular the secretion of gastrin, secretin, and VIP, delaying gastric emptying. While the tetradecapeptide somatostatin-14 is the prominent isoform in the hypothalamus, in the gut the larger molecule somatostatin-28 is more prevalent. Somatostatin exhibits pleiotropic actions thoughout the body, many of which involve the inhibition of secretion of several hormones, hence its name ‘endocrine cyanide’ (7).

Somatostatin has a wide range of therapeutic possibilities in different tissues; to capitalize on the diversity of its actions, many stabilized analogs and agonists have been synthesized over the years, extending the half life of somatostatin for sustained treatment effects (8). Octreotide is a first generation SRL, with a greater potency than native somatostatin for the specific somatostatin subtype 2 receptor and has a half-life of 90-120 minutes. While short-acting octreotide continues to be used in therapeutic practice in CHI, longer acting depot formulations have been developed, and are approved for the treatment of pituitary and neuroendocrine tumours (5, 9), but increasingly used in the treatment of CHI.

**Figure 2:**
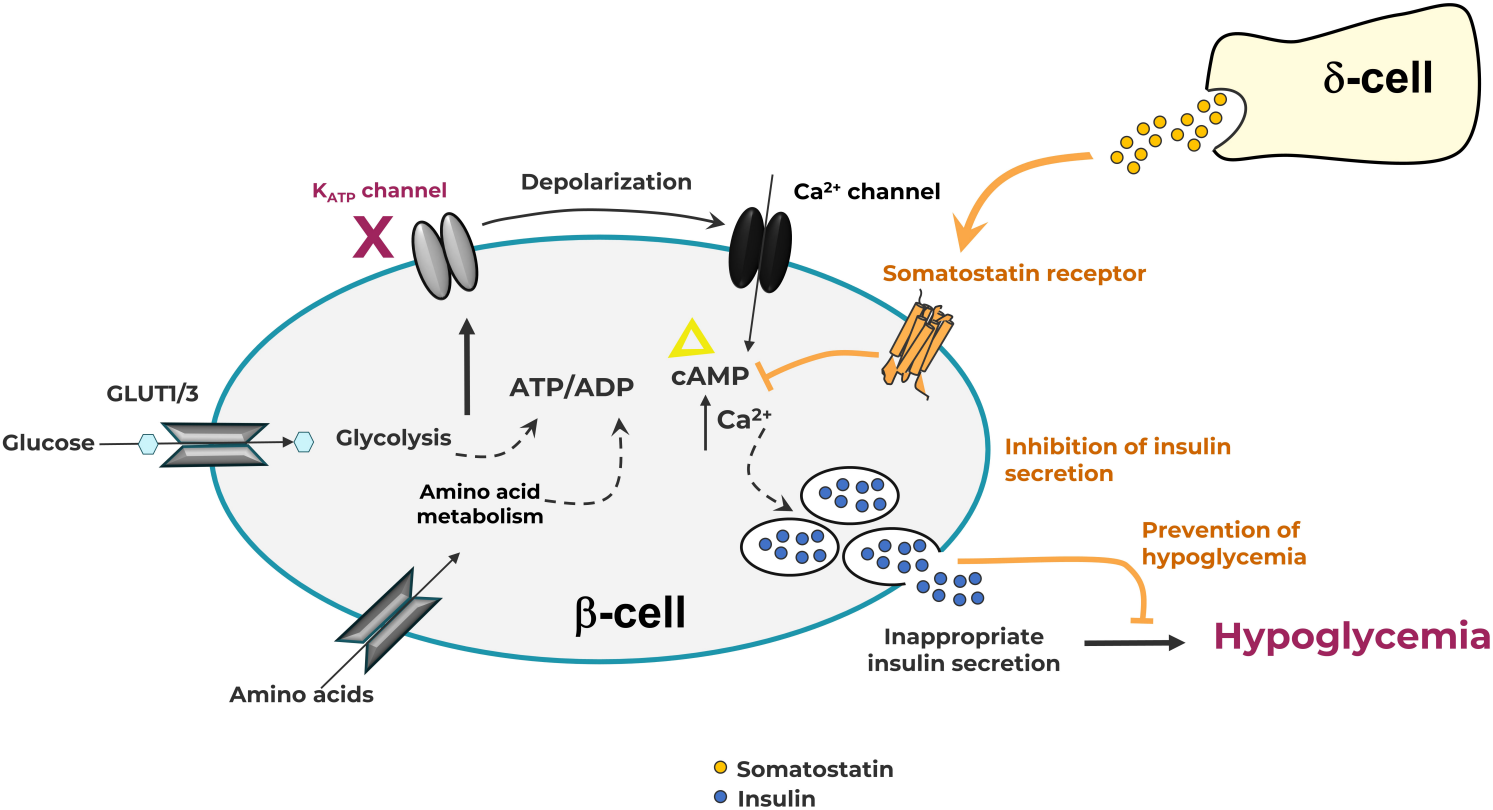
The pancreatic β-cell in Congenital Hyperinsulinism showing somatostatin action through G-protein coupled somatostatin receptors on the cell membrane. Elevation of intracellular ATP level drives K-ATP channel closure, membrane depolarization, and subsequent influx of Ca^++^ ions. Increases in intracellular Ca^++^ and cAMP levels lead to the release of insulin. Somatostatin receptor activation induces the formation of G_i_-GTP, which inhibits adenylate cyclase, preventing the formation of cAMP, thus reducing insulin secretion.

The wide array of effects of somatostatin is mediated by five somatostatin receptor (SST) subtypes (SST1-5), each encoded by a different gene (10). All somatostatin receptors are members of the class-A subgroup of the G-protein coupled receptor (GPCR) superfamily and activate G_i/o_ resulting in inhibition of adenylate cyclase and a decrease in cAMP levels. In response to agonists, SST2 is known to be phosphorylated by G protein receptor kinases (GRKs) and recruits β-arrestins resulting in receptor desensitization and internalization (11). These events and others trigger a range of additional downstream signaling and anti-proliferative effects. However, inhibition of the second messenger cAMP is the primary pathway responsible for its anti-secretory effects. SST2 on islet α-cells suppress glucagon (12), while SST2 and SST5 are primarily responsible for the suppression of insulin in β-cells (6) (**Figure 1**). In the β-cell, cAMP is hypothesized to be an amplifier of insulin secretion triggered by Ca^2+^ elevation (13); this process is targeted by SRLs to reduce insulin release (**Figure 2**). It is likely that the somatostatin actions are dependent on the density and distribution of SST receptor subtypes in different tissues (6) as well as variable receptor expression in CHI patients (14).

## Intra-islet actions of somatostatin

The human pancreas includes 1-3 million pancreatic islets (15) with a complex interplay between cell types. The secretion of insulin and glucagon by β-cells and α-cells varies reciprocally as a response to plasma glucose levels. Somatostatin-secreting δ-cells are the third most common cell type, representing ~5-10% of islet cells. They possess long neurite-like processes, which can interact with many α- and β-cells (3, 16) making them suitable candidates to exert paracrine regulation (**Figure 1**).

In isolated human islets, somatostatin is released in response to increasing glucose concentrations (17). The regulation of somatostatin secretion resembles that of insulin secretion from β-cells (18, 19). Like β-cells, δ-cells possess K-ATP channels that close with increasing glucose concentrations resulting in membrane depolarization and somatostatin secretion. These cells similarly respond to sulfonylureas, such as tolbutamide. Indeed diazoxide, which keeps the K-ATP channel complex in the open conformation to prevent depolarization and inhibit insulin secretion in β-cells, also inhibits somatostatin secretion from δ-cells (20). Like other pancreatic hormones, somatostatin is stored temporarily in secretory granules within δ-cells (21) and are released in response to perturbations in inhibitory and excitatory influences.

Endogenous somatostatin is regulated by intra-islet paracrine influences (22) and provides negative feedback to β-cells soon after glucose stimulated insulin release to prevent persistent insulin secretion in physiological states (23–26).

The emerging picture of healthy glucose-insulin secretion coupling is less an isolated β-cell driven action, but more a finely tuned paracrine counterbalance between all islet cell components. In CHI, many of the genes that are mutated in the β-cells of patients are also mutated in both α- and δ-cells. The net effect of the breakdown of this intra-islet homeostasis results in the dysregulation of insulin secretion, leading to significant hypoglycemia (26). More work is needed to unravel how the effects of α-cell and δ-cell dysfunction in CHI pancreatic islets contribute to the overall pathophysiology of CHI and determine individual phenotypes of disease.

## Somatostatin receptor distribution

A large number of studies have sought to characterize the expression of SSTs in human pancreatic tissues, but cataloging the differences between them is beyond the scope of this review (6, 12, 27, 28). Taken together, somatostatin effects in β-cells are predominantly mediated by SST2, SST5 and perhaps SST1, while the impact of SST3 is unclear. SST4 does not appear to have functional expression in the pancreas. In the α-cell, most histological, expression and functional data points to the conclusion that SST2 is the dominant receptor.

Recent work from islets isolated from CHI patients undergoing pancreatectomy for focal, diffuse, or anomalous CHI (29) suggested less somatostatin activity than matched controls (30). Intriguingly, SST2 was expressed in nearly all CHI patients, while SST5 was expressed less frequently, although one patient with diffuse CHI did not express either SST2 or SST5 (29). This observation needs to be replicated in other cohorts to understand the real frequency of SST receptor expression variation and the potential implication of selective SST expression for optimal treatment of patients. Further, SST receptor downregulation mechanisms need to be explored, with initial work (14) suggesting a role for treatment related expression variability in non-focal CHI.

## Somatostatin receptor ligand use in congenital hyperinsulinism

Somatostatin treatment was first described in a child with CHI after 80% pancreatectomy (31), followed by use of a continuous subcutaneous infusion *via* a pump in a 6-month old infant with insulin excess (32). Following initial demonstration of effect and the synthesis of compounds with extended activity, SRLs are now available for many patients with CHI unresponsive to diazoxide. The short acting SRL octreotide has been used in the treatment of CHI (33, 34) as second line therapy over the last two decades to prevent hypoglycemia and subtotal/near-total pancreatectomy. Currently, SRL therapy is not generally used as first line treatment, except in situations where diazoxide treatment is contraindicated or in countries where diazoxide is not readily available.

### Short acting SRL treatment

The strategy to use octreotide subcutaneous injections administered every 4 to 8 hours (35) or as continuous infusions (36) brought some benefit in early observational studies, but response to doses up to 40 mcg/kg/day were not clinically effective to prevent pancreatectomy in the majority of patients. However, following wider use, reports of improved outcomes and avoidance of pancreatectomy were noted (37, 38), establishing octreotide as standard, albeit off-label therapy for CHI. Octreotide is now commonly used as subcutaneous bolus injections or by continuous subcutaneous infusions (using insulin pumps) in doses typically ranging from 5 to 40 microgram/kg/day (39). While the use of higher doses have been reported, treatment effect is rare beyond 20 micrograms/kg/day in most patients. For patients requiring frequent injections or higher doses, short-acting octreotide may be substituted by long acting SRL formulations such as octreotide long acting release (e.g. Sandostatin™, Olatuton™) or lanreotide autogel (Somatuline™).

### Long acting SRL treatment

Long acting SRLs have been tried in small groups of patients with reported success (40, 41) in obervational studies. They have the advantage of reduced frequency of administration and therefore the potential to improve patient quality of life (42). However, depot injection can be painful and inefficiently dosed, even when administered by trained staff (43). A number of observational studies have reported on different markers of efficacy (44, 45), but in the absence of a comparative or control arm, the efficacy of such long acting SRLs cannot be calibrated to meaningful outcomes such as the achievement of normoglycaemia and harm-free survival. Further, objective assessments of short and long acting SRLs have not been undertaken to appreciate comparative benefits and risks, given the repurposing focus for clinical use outside standardised trial protocols.

### Side effects of SRL treatment

Both octreotide and lanreotide possess similar SST receptor pharmacological profiles (primarily SST2 agonists) (**Supplementary Table 1**) and are therefore expected to have a similar range of both therapeutic and adverse effects. The long acting depot preparations can persist in tissues for up to a month or longer. In young children this might lead to cumulative effects although the extent of accumulation of adverse effects is not known (45).

The utility of octreotide and other SST2 agonists are often complicated by loss of effect with increasing dose in the initial phase of treatment. This downregulation of the SRL dose-response curve is likely to be a consequence of receptor desensitization and internalization, although demonstration of this effect has not been shown in CHI pancreatic tissue. Octreotide and other SRLs have a number of adverse effects including the prolongation of gastrointestinal transit time, abdominal discomfort, and fat malabsorption (46), which could add to feeding problems or require treatment with pancreatic enzymes. Biliary sludging and accumulation into stones (47) are reported in adults (46) and children (48) although the need for cholecystectomy has not been described.

SRL therapy has been used for many years to reduce growth hormone excess, mainly in adults. As SRL treatment also reduces growth hormone secretion in children, regular auxology follow up in CHI patients is required. While the incidence of growth hormone deficiency following octreotide remains low (47), it is possible and perhaps likely, that this side effect is under-reported in the absence of long-term auxology datasets in CHI cohorts. As growth is an essential component of childhood, careful examination of stature and organ growth needs to be undertaken with the increasing use of long acting SRLs in CHI. Octreotide use in pregnant mothers with CHI also predisposes the fetus to growth restriction, but may represent the only therapeutic option (49) as alternatives such as diazoxide cause unacceptable reduction in placental blood flow.

In newborn babies, particularly those preterm, the risk of necrotizing enterocolitis, possibly arising from reduced splanchnic vascular flow, is significant and can be life threatening (50, 51). Such risk persists beyond the neonatal period, thus necessitating careful review in follow-up of all patients. Although not life threatening, all forms of short and long acting SRLs have the propensity to cause hepatitis (45, 52), an adverse effect that precludes long-term use in the absence of data demonstrating normal hepatic outcomes in later life. Less commonly reported adverse effects include pancreatic exocrine insufficiency (53) and long QT syndrome with potential risk for cardiac arrythmias (54). Octreotide can also cause drug induced pancreatitis (55), an effect that needs to be heeded through stepped down withdrawal of treatment prior to pancreatectomy in CHI patients. Although not widely reported in CHI, octreotide can cause hair loss (56), an effect that might seem trivial but could have significant impact on the psychosocial well-being of older children.

## Emerging somatostatin receptor ligands

Several SRLs have been utilised in the treatment of CHI, mainly in those are unresponsive and in those who experience adverse effects from diazoxide treatment. On the whole there is treatment benefit (45) although quantification is imprecise and probably unreliable. Further, loss of initial treatment effect often seen with octreotide is also likely to be present with long acting SRLs, causing later recurrence of hypoglycaemia.

It is likely that SRLs in development for other conditions such as acromegaly [e.g. paltusotine, https://clinicaltrials.gov/ct2/show/NCT04837040] may also be repurposed for use in CHI patients. An example of such a SRL is pasireotide, currently FDA-approved for the treatment of Cushing’s disease in adults. In clinical trials in Cushing’s disease, treatment with pasireotide resulted in increases in glucose levels, suggesting collateral pancreatic effect (57). Pasireotide possesses a greater potency for stimulated cAMP inhibition of SST5 than octreotide or lanreotide while also possessing some potency for SST2 and SST3 (58) (**Supplementary Table 1**). Pasireotide has been observed to result in less inhibition of glucagon secretion than octreotide (57), an effect that may be beneficial in the counterregulatory response to hypoglycaemia in CHI patients. Schwetz et al. (59) described the use of pasireotide effectively to control persistent hypoglycemia in an adult patient with CHI like features. Mooij et al. (60) reported on the use of both short-acting pasireotide injections and long-acting pasireotide in an infant with homozygous *ABCC8* mutations; hypoglycaemia frequency improved but was not sufficient to prevent near-total pancreatectomy. Similar to the off-label use of other long acting SRLs, the long term treatment benefit of pasireotide remains to be clarified.

The use of off-label SRLs has prompted the development of new agonists targeting specific SST receptors, in particular SST5. A First in Human study to assess the safety, tolerability, PK, and PD of HTL0030310 compared to pasireotide has been registered in 2019 [https://www.clinicaltrials.gov/ct2/show/NCT03847207] but results are not yet available.

An orally-available selective nonpeptide SST5 agonist, CRN02481, has been shown to suppress insulin secretion and increase glucose levels in both oral glucose tolerance tests and a sulfonylurea model of hyperinsulinism (61, 62). CRN02481 prevented fasting hypoglycaemia and amino acid-stimulated insulin secretion in a Sur1^-/-^ mouse model of CHI (63). Moreover, CRN02481 significantly decreased insulin secretion in human islets isolated from two patients with CHI and one patient with Beckwith-Weideman Syndrome (BWS) CHI, providing ex-vivo demonstration of efficacy in targeted hyperinsulinaemic patients (63).

## Conclusions

The biology of SST receptors and ability of SRLs to activate specific receptor subtypes to reduce excess insulin is important in the understanding of the pathobiology and treatment perspectives in CHI. Pharmacological targeting of SST receptors reduces insulin release directly, bypassing K-ATP channel defect dysregulation, thereby providing treatment alternatives to diazoxide-unresponsive CHI patients. Both short and long acting SRLs have been used as second line treatments with similar therapeutic and adverse effect profiles. SRLs with specific β-cell action are currently being developed; these drugs may have improved efficacy and reduced adverse effect profiles, providing much needed therapeutic choice before considering irreversible sub-total pancreatectomy as a last resort for the treatment of severe and recurrent hypoglycaemia.

## Author contributions

All authors contributed to the first draft of the manuscript. All authors contributed to the manuscript revision. MA, KM, IB and SB read and approved the submitted version. To our sorrow, MD unexpectedly died during the drafting phase of this manuscript.

## Conflict of interest

Author IB is associated with research grants in the development of somatostatin receptor ligands. He is also involved in novel drug development in Congenital Hyperinsulinism. Author SB is an employee of Crinetics Pharmaceuticals, which has an interest in the development of specific forms of somatostatin receptor ligands in Congenital Hyperinsulinism.

The remaining authors declare that the research was conducted in the absence of any commercial or financial relationships that could be construed as a potential conflict of interest.

## Publisher’s note

All claims expressed in this article are solely those of the authors and do not necessarily represent those of their affiliated organizations, or those of the publisher, the editors and the reviewers. Any product that may be evaluated in this article, or claim that may be made by its manufacturer, is not guaranteed or endorsed by the publisher.
