# Supplementary material for: Somatostatin receptors in congenital hyperinsulinism: Biology to bedside
Source: Front Endocrinol (Lausanne). 2022 Sep 27;13:921357. doi: 10.3389/fendo.2022.921357 (PMC9552539; doi:10.3389/fendo.2022.921357)
Supplement: Supplementary file 1 [file Table_1.docx]

**Supplementary Table 1**

|  | SST1 | SST2 | SST3 | SST4 | SST5 |
| --- | --- | --- | --- | --- | --- |
| Islet cell somatostatin receptor expression | | | | | |
| α-cell | - | ++ | -- | -- | +/- |
| β-cell | +/- | ++ | +/- | -- | ++ |
